# Supplementary material for: Natural Genetic Variation Impacts Stress-Induced Quiescence and Regeneration in Response to Rapamycin
Source: Cells. 2026 Jan 26;15(3):236. doi: 10.3390/cells15030236 (PMC12896840; doi:10.3390/cells15030236)
Supplement: Supplementary file 1 [file cells-15-00236-s001.zip › cells-4041938-supplementary/Supplemental Figure S1.pdf]

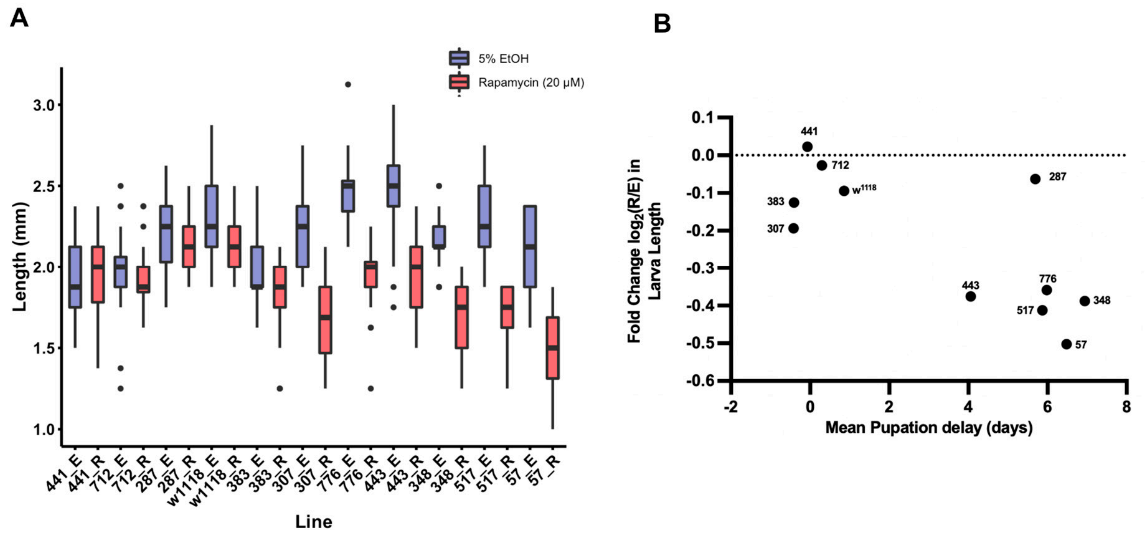

Supplemental Figure S1. (A) Length (mm) of larvae at 72 h post-oviposition, treated with yeast paste dissolved with 5% EtOH (solvent control) or rapamycin (20 μM) diluted in 5% EtOH (see Methods). Red-shaded box-plots correspond to lengths from rapamycin-treated larvae and blue-shaded box-plots correspond to lengths from control-treated larvae (n = 19-62 larva). Batches were repeated if n<15. (B) Sensitivity to rapamycin in larva length represented as a log_2 fold change of mean length (rapamycin/control) over the sensitivity to rapamycin in pupation delay (days, Harrison et al., 2024), where Spearman's correlation (r_s = -0.655, P-value = 0.029).
