# Supplementary material for: Natural Genetic Variation Impacts Stress-Induced Quiescence and Regeneration in Response to Rapamycin
Source: Cells. 2026 Jan 26;15(3):236. doi: 10.3390/cells15030236 (PMC12896840; doi:10.3390/cells15030236)
Supplement: Supplementary file 1 [file cells-15-00236-s001.zip › cells-4041938-supplementary/Supplemental Figure S2.pdf]

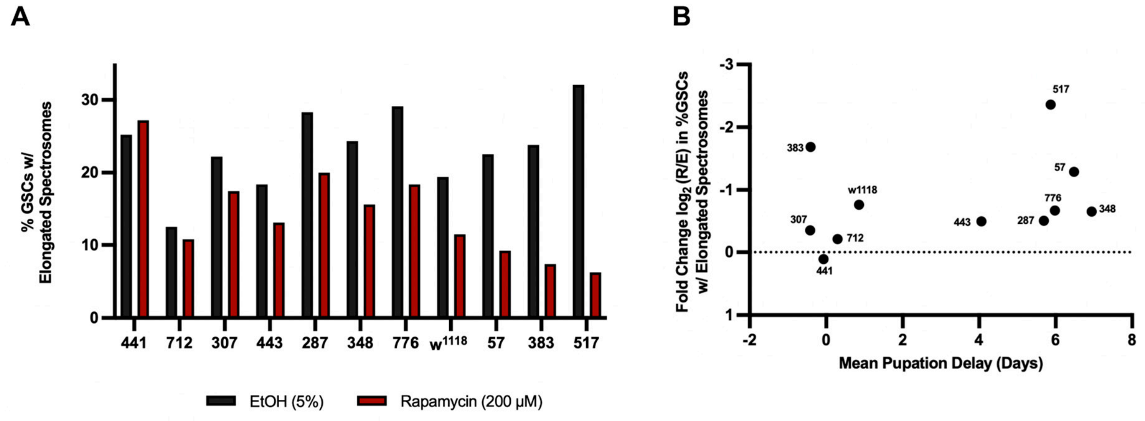


Supplemental Figure S2. (A) Percentage of GSCs w/ elongated spectrosomes in each DGRP line and $w^{1118}$ at 2 dpi treated with rapamycin (200 μM) or 5% EtOH (solvent control). Red corresponds to data for rapamycin-treated flies, and black corresponds to data for control-treated flies (n=158-212 GSCs). (B) The sensitivity to rapamycin in delay in cell-cycle reentry represented as a $\log_2$ fold change of % GSCs with elongated spectrosomes (rapamycin/control) over the sensitivity to rapamycin in developmental delay (days, Harrison et al., 2024), where Spearman's correlation ($r_s = 0.4$, P-value = 0.223).
